# Toward precision hernia surgery: integrating biomarkers, genomics, quantitative imaging, and artificial intelligence in abdominal wall repair

**DOI:** 10.1007/s10029-026-03864-8

**Published:** 2026-10-01

**Authors:** Bruno Amantini Messias, Guilherme Costa e Silva, Pedro Henrique de Freitas Amaral, Diogo Parente Falcão, Sergio Roll, Jaques Waisberg

**Affiliations:** 1 Servidor Publico Estadual Hospital (IAMSPE), 1800, Pedro de Toledo Street, São Paulo, 04039-000 Brazil; 2 Samaritano Hospital, 1486, Conselheiro Botero Street, São Pulo, 01232-010 Brazil; 3 Santa Casa of São Paulo, 112, Cesario Mota Junior Street, São Paulo, 01221-020 Brazil; 4 Sao Marcelino Champagnat Hospital, 1399, Presidente Affonso Camargo Avenue, Curitiba, 80050-370 Brazil

**Keywords:** Hernia, Abdominal, Extracellular matrix, Inflammation, Fibroblasts, Mesh, Precision medicine

## Abstract

**Purpose:**

Despite technical advances, the expansion of minimally invasive approaches and the development of novel biomaterials, recurrence and complications following abdominal wall hernia (AWH) repair remain frequent. This variability likely reflects anatomical and technical factors and host biological heterogeneity. Precision herniology is emerging as a translational paradigm integrating biomarkers, genetics, quantitative imaging, and artificial intelligence to refine risk stratification and therapeutic planning.

**Methods:**

We conducted a critical narrative review with translational scope. PubMed/MEDLINE, Scopus, Web of Science, and Embase were searched from January 2015 to June 2026. We prioritized adult studies across four axes: (i) biomarkers of collagen, extracellular matrix, and cellular mechanisms; (ii) genetics and susceptibility to AWH; (iii) immunonutritional markers; (iv) artificial intelligence (AI) and machine learning (ML) for predicting complexity and complications.

**Results:**

The literature reveals convergent, albeit heterogeneous, signals indicating that host biology may contribute to variability in hernia phenotype and surgical outcomes. Alterations in collagen turnover and in the MMP-TIMP axis, fibroblast heterogeneity, inflammatory signaling, polygenic architecture related to connective tissue integrity, immunonutritional markers associated with perioperative vulnerability, and quantitative imaging metrics analyzed by AI/ML models represent biologically plausible, clinically relevant domains.

**Conclusion:**

Available data support the plausibility that host biological heterogeneity contributes to the formation, healing, prosthetic integration, and recurrence of AWH, thereby broadening the traditional paradigm centered predominantly on defect anatomy. The main value of this work is the critical integration of molecular and cellular biomarkers, genetics, quantitative imaging, and AI/ML into a unified conceptual framework for future precision herniology research.

**Supplementary Information:**

The online version contains supplementary material available at https://doi.org/10.1007/s10029-026-03864-8.

## Introduction

Abdominal wall hernias (AWH) represent a relevant surgical and socioeconomic challenge. More than 20 million inguinal hernia repairs are performed worldwide each year [1]. For ventral hernias in the United States, operations exceeded 600,000 repairs/year, with an aggregate cost of US$9.7 billion [2]. Despite this burden, contemporary guidelines remain largely focused on operative technique, prosthetic positioning, approach selection, and complication prevention [3–5], while incorporating individualized host biology only to a limited extent.

Despite minimally invasive approaches (laparoscopic/robotic) and next-generation meshes, recurrence rates remain high, particularly in ventral and incisional hernias. In a contemporary analysis of the Abdominal Core Health Quality Collaborative registry, the 5-year cumulative clinical recurrence after ventral hernia repair was 44.9% among patients who underwent mesh repair and 73.7% among those who underwent non-mesh repair [6]. Chronic pain, wound occurrences, and infection remain clinically important outcomes, are discussed centrally in guidelines, and directly affect postoperative results [4, 5, 7]. Together, recurrence, pain, and surgical site occurrence remain major determinants of outcomes after AWH repair [4–7].

The abdominal wall is a dynamic biomechanical structure with substantial interindividual variation, as demonstrated by dynamic MRI during breathing, coughing, and Valsalva maneuvers [8]. Its integrity depends on ECM remodeling, particularly fibrillar collagens I and III, whose imbalance may reduce connective-tissue strength and contribute to abdominal wall vulnerability. Evidence from fascial and non-fascial tissues suggests that this dysfunction may extend beyond the hernia defect, while MMP-mediated matrix degradation provides a mechanistic link between collagen dysregulation and tissue weakening [9–11]. In parallel, GWAS data support genetic susceptibility involving pathways related to connective-tissue structure, ECM organization, collagen, and elastin homeostasis [12].

Nutritional status also directly influences surgical morbidity and wound healing. Perioperative nutrition guidelines recommend systematic screening for nutritional risk and preoperative optimization, particularly in high-risk patients, because malnutrition increases the risk of postoperative complications [13]. Similarly, computed tomography (CT)-based body composition assessment in complex abdominal wall surgery showed high mortality in sarcopenic patients, reinforcing nutritional assessment and optimization as a pillar of perioperative care in AWH [14].

Precision medicine offers a paradigm for AWH management, proposing individualized decisions based on patient and disease characteristics. Broadly, it incorporates biological, clinical, behavioral, and environmental data to guide more personalized prevention and treatment [15]. In abdominal wall surgery, individualized repair tailored to the patient’s risk profile and defect characteristics has maintained uniformly low perioperative complication rates, including in higher-risk patients [16].

Artificial intelligence tools, including machine learning, large language models, and computer vision, are increasingly being explored in hernia surgery for risk prediction, assessment of surgical complexity, intraoperative support, and refinement of minimally invasive abdominal wall reconstruction [17, 18].

In parallel, biomechanical studies show that the abdominal wall has region-specific composition, sex-related differences in thickness and stiffness, and anisotropic properties that are not fully reproduced by many meshes. Because many recurrences occur at the biomaterial–tissue interface rather than by material rupture, mesh orientation, positioning, and host–implant mechanical compatibility may be as relevant as tensile strength alone [19].

We hypothesize that, in a subset of patients, hernia repair failure reflects not only technique, mesh position, or defect characteristics, but also host biological heterogeneity, including collagen/ECM turnover, inflammatory and fibroblast responses, the proteolytic balance between metalloproteinases and their inhibitors, genetic predisposition, and immunonutritional vulnerability, which may influence healing, prosthetic integration, and the inflammatory response. This hypothesis is biologically plausible, but its clinical relevance likely varies across hernia subtypes, operative contexts, and outcomes.

In this study, we critically explored how the integration of cellular and molecular biology, circulating biomarkers, genomics, immunonutrition, quantitative imaging, biomaterial–host interactions, and AI/ML could support more individualized risk stratification in AWH. The future of herniology will not be defined by how we close the defect, but also by how early we can recognize the host biology that conditions repair durability.

## Methods

This is a critical narrative review with a translational scope, integrating clinical, laboratory, imaging, and digital evidence on the pathophysiology and management of AWH within precision medicine. We aimed to map and critically analyze domains with translational relevance, biomarkers, genetics, immunonutritional markers, quantitative imaging, and AI/ML, and to identify the main gaps limiting their integration into clinical risk stratification. PubMed/MEDLINE, Scopus, Web of Science, and Embase were searched from January 2015 to June 2026. The search combined MeSH descriptors and free-text terms using Boolean operators, focusing on the main thematic axes. Combinations included (“abdominal wall hernia” OR “ventral hernia” OR “inguinal hernia”) AND (collagen OR “extracellular matrix” OR “type I collagen” OR “type III collagen” OR PINP OR PICP OR PIIINP OR PIIICP OR MMP OR TIMP); (“abdominal wall hernia”) AND (genetic OR polymorphism OR “precision medicine” OR “risk stratification”); and (“abdominal wall hernia”) AND (“artificial intelligence” OR “machine learning” OR “prediction model” OR “outcome prediction”); and (“abdominal wall hernia”) AND (inflammation OR macrophage OR fibroblast OR myofibroblast OR cytokine OR “growth factor” OR “foreign body reaction” OR “mesh integration”). Additional studies were identified via manual searching of the reference lists of selected articles.

We prioritized studies with greater translational relevance, including society guidelines, systematic reviews, multicenter studies, large-scale genomic analyses, and clinical investigations with outcomes related to AWH formation, recurrence, or complications. Mechanistic and exploratory studies were included when they supported biological plausibility or clarified relevant research gaps. Prioritization also considered design robustness, proximity to clinically relevant outcomes, consistency of findings, applicability to AWH, and translational maturity of the domain.

Title and abstract screening and full-text review were performed by two independent reviewers, with discrepancies resolved by discussion. For each study, we extracted design, year, population, hernia subtype (inguinal, ventral, or incisional), biomarkers or genetic variables, cellular and inflammatory variables, immunonutritional markers, clinical outcomes, and main limitations. For AI/ML studies, we also recorded input data, model type, performance metrics, and validation strategy.

The synthesis was descriptive, critical, and integrative, structured into four axes: (i) biomarkers of collagen, extracellular matrix and cellular mechanisms; (ii) genetic markers; (iii) immunonutritional markers; and (iv) digital tools, with emphasis on AI/ML. Within each axis, we distinguished mechanistic plausibility, initial translational signal, and clinical readiness, with attention to methodological limitations and heterogeneity. No formal risk-of-bias assessment was performed, as the review aimed not to estimate pooled effects or compare interventions but to critically synthesize emerging, translationally relevant domains. For the same reason, no meta-analysis was performed, given substantial heterogeneity in populations, hernia subtypes, biological matrices, assays, analytical platforms, outcome definitions, and follow-up. We therefore adopted an integrative approach focused on patterns, convergences, gaps, and research directions rather than quantitative effect estimates. The conclusions should be interpreted as a critical synthesis and proposed translational agenda, rather than consolidated evidence for immediate clinical implementation.

## Results

The results are presented as a structured narrative synthesis. The four axes identified showed unequal translational maturity. Collagen/ECM turnover, cellular mechanisms and genetics offer strong biological plausibility but limited clinical validation; the immunonutritional axis represents a modifiable layer of perioperative vulnerability; and quantitative imaging with AI/ML is closer to predictive use, though it still requires interpretability and external validation.

### Molecular and cellular markers: collagen metabolism, ecm remodeling, and cellular mechanisms

An imbalance between type I and type III collagen is common in the abdominal wall of patients with hernias. Type I collagen predominates in high-tensile-strength tissue, and type III collagen confers compliance and elasticity; their proportions are altered in these patients. A reduced collagen I/III ratio has been reported in primary and recurrent hernias and is associated with matrix and healing alterations, as well as a higher recurrence risk [9–11]. Additional evidence implicates type IV collagen turnover in basement membrane remodeling and type V collagen in fibrillogenesis and tissue micromechanics, with population-based data linking fibrillar collagenopathies to increased inguinal hernia susceptibility [20–23].

#### Synthesis markers

During collagen biosynthesis, amino- and carboxy-terminal propeptides are cleaved from procollagen extracellularly and released into the extracellular milieu, where they may reach the circulation. Among these, PIIINP (type III collagen amino-terminal propeptide) and type I collagen propeptides (such as PINP/PICP) are particularly relevant, along with PIIICP. As with all procollagen propeptides, these fragments are enzymatically cleaved before fibril assembly and are not incorporated into the mature collagen fibers; therefore, they reflect synthetic and turnover activity rather than fiber content. Recent multiplexed targeted proteomics enables simultaneous detection of these propeptides (e.g., PINP, PICP, PIIINP, and PIIICP), providing a robust tool to dynamically investigate collagen metabolism and tissue remodeling [24].

Clinical and translational studies indicate that circulating collagen propeptides, particularly type I procollagen N-terminal peptides (PINP), PIIINP, and the PINP/PIIINP ratio, reflect altered collagen synthesis and turnover in patients with inguinal hernia and may be associated with recurrence [25, 26]. The carboxy-terminal propeptide of type I collagen (PICP), a marker of type I collagen synthesis, may further help characterize matrix anabolic–catabolic balance when combined with other ECM markers [24]. Together, these findings, supported by reviews and recent evidence on ECM remodeling, oxidative stress, and collagen metabolism in inguinal and incisional hernias, suggest that reduced fibrillar collagen synthesis and broader ECM turnover signatures may serve as candidate biomarkers of tissue vulnerability and recurrence risk in AWH; however, analytical standardization and prospective validation remain necessary [20, 27, 28] (Table 1).

**Table 1:** Characteristics and clinical relevance of the main collagen propeptides

Biomarker	Collagen type	Propeptide region	Clinical significance
PINP	Type I	N-terminal	Type I collagen synthesis (reflects fibrillar matrix formation)
PICP	Type I	C-terminal	Type I collagen synthesis (released during collagen maturation)
PIIINP	Type III	N-terminal	Type III collagen synthesis (reflects connective tissue remodeling)
PIIICP	Type III	C-terminal	Type III collagen synthesis (quantified indirectly, analytically emerging)

PINP N-terminal propeptide of type I procollagen, PICP C-terminal propeptide of type I procollagen, PIIINPN-terminal propeptide of type III procollagen, PIIICP C-terminal propeptide of type III procollagen

#### Degradation markers

Type I collagen degradation markers, such as ICTP (C-terminal telopeptide) and NTX (N-terminal telopeptide), are fragments of type I collagen cleavage products that reflect fibrillar matrix resorption, usually quantified by standardized immunoassays (e.g., ELISA/CLIA) [29]. MMP-generated neoepitopes, such as C1M (collagen I) and C3M (collagen III), more specifically reflect interstitial ECM degradation and can be detected by targeted immunoassays [30]. In patients with AWH, an altered serum collagen turnover profile is observed, involving synthesis markers (including PINP) and degradation markers (including C1M/C3M) [30].

#### ECM regulators

Matrix metalloproteinases (MMPs) degrade ECM components such as collagen, elastin, and proteoglycans; their activity is modulated by tissue inhibitors of metalloproteinases (TIMPs), preserving the balance between remodeling and degradation. MMP-TIMP imbalance has been documented in tissue and serum from patients with inguinal and incisional hernias, supporting inadequate abdominal wall remodeling [31, 32]. In particular, immunohistochemical analyses of abdominal wall tissue showed alterations in TIMP-1 and TIMP-2 in patients with hernia (with TIMP-1 more highly expressed in hernia groups and TIMP-2 more highly expressed in controls) [31]. In addition, recent serum studies demonstrated increased MMP-1 and net MMP activity (MMP/TIMP ratios, such as MMP-1/TIMP-1 and MMP-1/TIMP-2) in patients with inguinal hernia, reinforcing a more catabolic ECM phenotype [32].

Pre- and postoperative comparison shows that MMPs/TIMPs vary over time, reinforcing the dynamic nature of remodeling (postoperative changes in MMP-2/MMP-9 and TIMP-1/TIMP-2) [33]. Thus, serum and/or tissue quantification of MMPs/TIMPs may serve as an integrated indicator of catabolic activity and matrix remodeling in AWH [31–33].

#### Inflammatory and cellular mechanisms

Recent human studies support the involvement of inflammatory and stromal-cell dysregulation in abdominal wall hernia, although the available evidence remains predominantly exploratory. In incisional-hernia tissues, fibroblast-associated markers differed between the hernia ring and sac: CTGF expression was increased, and α-smooth-muscle actin was reduced in both compartments, whereas CD34, FSP1, and cadherin-11 showed compartment-specific patterns, supporting fibroblast heterogeneity within established hernia tissue [34]. In inguinal-hernia specimens, IL-1β expression was increased and positively correlated with MMP-2. Complementary experiments in human skin fibroblasts demonstrated that IL-1β activated p38 MAPK signaling, increased MMP-2 expression, impaired cell proliferation and migration, and altered the collagen I/III ratio [35].

Human polypropylene mesh explants removed primarily because of recurrence exhibited a spatially concentrated macrophage response near the mesh fibers. Approximately 60% of macrophages expressed M2-associated markers, whereas approximately 6% expressed an M1-associated marker; macrophages also colocalized with collagen I or III and MMP-2, indicating that remodeling-associated macrophage phenotypes may coexist with collagen deposition and MMP-2-associated remodeling [36]. Analysis of the same seven explants further demonstrated that both innate and adaptive immune cells participated in the chronic foreign-body response, with macrophages, T-helper cells, and regulatory T cells concentrated near the mesh interface [37].

At the systemic level, Mendelian randomization analysis identified associations between genetically predicted inflammatory mediators, immune-cell traits, and incisional-hernia susceptibility, including an association of IL-5 with increased risk and TNF-related activation-induced cytokine with a lower risk [38]. A bidirectional Mendelian randomization study found no FDR-significant evidence that circulating inflammatory cytokines causally increased ventral-hernia risk; conversely, genetic liability to ventral hernia was associated with higher circulating macrophage inflammatory protein-1β levels, a finding consistent with—but not sufficient to establish—a downstream inflammatory consequence of the disease [39].

However, these genetic findings do not clarify the local tissue mechanisms. Collectively, current human evidence aligns with fibroblast heterogeneity and inflammatory–proteolytic signaling in abdominal wall hernia, as well as a complex macrophage and lymphocyte response at the mesh–tissue interface. However, small tissue cohorts, evaluation predominantly after disease establishment, overlap between mesh-explant samples, and the absence of prospective validation preclude causal inference or the use of cellular phenotypes for clinical risk stratification.

#### Emerging glycomic biomarkers

Emerging omics approaches may complement conventional serum markers of collagen turnover and ECM remodeling by expanding biological characterization in hernia research. In inguinal hernia, exploratory serum N-glycome profiling identified altered glycan traits related to fucosylation and linkage-specific sialylation, suggesting that systemic post-translational glycoprotein signatures may contribute to a broader multi-omic framework for precision herniology, although external validation remains necessary [40].

#### Exploratory plasma proteomic biomarkers

In the ColoCare cohort, antibody-microarray profiling of preoperative plasma from 72 patients undergoing midline colorectal cancer surgery identified 25 proteins with nominally different levels in patients who subsequently developed incisional hernia, including candidates related to wound healing (CCL21, SHBG, and BRF2) and cell adhesion (PCDH15, CDH3, and EPCAM); however, these discovery-phase findings require replication in independent cohorts and clinical validation [41].

### Genetic markers: the underlying predisposition

AWH susceptibility has a polygenic basis, with GWAS identifying multiple loci enriched in pathways related to ECM organization and connective-tissue stability [12]. Complementary TWAS and fascial transcriptomic studies suggest that regulatory variants and altered gene expression in abdominal wall tissues may contribute to hernia risk through ECM remodeling pathways and pleiotropic mechanisms across hernia subtypes [42, 43].

#### Genes and loci predisposing to hernias

Genomic studies reinforce the polygenic basis of hernia susceptibility. Early GWAS identified susceptibility loci near EFEMP1, WT1, EBF2, and ADAMTS6, supporting the involvement of connective-tissue pathways in inguinal hernia predisposition [44]. Subsequent multiethnic and population-specific studies expanded this genetic architecture, identifying additional ancestry- and sex-specific signals, including regions near LYPLAL1/SLC30A10, STXBP6/NOVA1, MYO1D, ZBTB7C, VCL, FAM9A/FAM9B, WT1, and EFEMP1 [45, 46]. Population-based data further link fibrillar collagenopathies, including COL5A1/COL5A2-related disorders, to increased inguinal hernia risk [23]. More recent genetic-correlation, Mendelian randomization, and proteomic analyses suggest shared genetic architecture and pleiotropy across hernia subtypes, prioritizing candidates such as BMP6, MYCBPAP, and ZNF75A for future validation [47].

#### Growth factor and stromal signaling

Growth-factor pathways may serve as a biological link between genetic susceptibility and stromal remodeling in abdominal wall hernia. A trans-ethnic meta-analysis combining BioBank Japan and UK Biobank data identified a genome-wide significant inguinal-hernia susceptibility locus near TGFB2; however, this genetic association does not establish altered local TGF-β2 expression or activity [48]. In established incisional-hernia tissues, CTGF expression was increased, and α-smooth-muscle actin expression was reduced in both the hernia ring and sac, while other fibroblast-associated markers showed compartment-dependent patterns [34]. More recently, single-nucleus multi-omic analysis of herniated lower abdominal muscle in a murine model identified Pgr-expressing fibroblasts associated with TGF-β2-mediated fibrosis and myofiber atrophy. Targeted analyses of paired human tissues subsequently demonstrated increased TGFB2 and TGFBR2 expression in herniated muscle compared with adjacent healthy muscle [49]. Collectively, these findings support the involvement of TGF-β2-related and CTGF-associated stromal remodeling in selected hernia phenotypes; however, differences in species, hernia subtype, tissue compartment, and study design preclude a unified causal interpretation or clinical application.

#### Emerging genomic and multi-omic mechanisms

Recent multi-omic studies suggest that hernia susceptibility may extend beyond isolated ECM candidate genes toward metabolic, hormonal, stromal, and tissue-specific mechanisms. Lipidomic Mendelian randomization implicates lipid-related metabolic pathways in inguinal hernia susceptibility, while experimental and human multi-omic data suggest that fibroblast progesterone receptor signaling, skeletal muscle fibrosis, myofiber atrophy, and TGF-β2–related stromal remodeling may contribute to inguinal hernia formation [49, 50].

#### Microbiome–genetic and network-based mechanisms

A bidirectional two-sample Mendelian randomization study using MiBioGen and FinnGen summary statistics reported nominal inverse associations between genetically predicted abundance of eight gut microbial taxa and inguinal-hernia susceptibility. Reverse analyses have identified nominal associations between genetic liability to inguinal hernia and six microbial taxa [51]. Because 196 taxa were evaluated and the reported associations were based on a nominal significance threshold, these findings should be considered exploratory and do not establish direct microbial mechanisms or clinical utility.

Separately, an integrative bioinformatics study combined text mining, protein–protein interaction networks, functional enrichment, drug–gene interaction mapping, and two-sample Mendelian randomization. The network analyses identified 37 candidate hub genes and showed enrichment of several signaling pathways, including PI3K–Akt, MAPK, AGE–RAGE, and HIF-1. The Mendelian randomizationcomponent further highlighted a subset of genes as potentially associated with inguinal hernia, with nine genes specifically emphasized by the authors. These findings may support earlier risk stratification and further investigation of the molecular mechanisms underlying inguinal hernia [52]. However, these predominantly computational findings should be considered hypothesis-generating. Replication using appropriately corrected multiple-testing thresholds, independent genetic datasets, functional validation in biologically relevant abdominal wall tissues, and prospective clinical studies will be required before these signals can be interpreted as mechanistically or clinically relevant.

### Immunonutritional markers

Nutritional status directly influences tissue healing, immune response, and prosthetic integration in abdominal wall surgery. Protein-energy malnutrition, often reflected by preoperative hypoalbuminemia, is associated with worse postoperative outcomes; more wound-related complications, including dehiscence; and higher morbidity and mortality [53].

#### Protein indicators

Albumin, prealbumin, and transferrin can be used in perioperative nutritional assessments; however, albumin is a negative acute-phase reactant and should also be interpreted as a prognostic marker influenced by inflammation [53]. Preoperative hypoalbuminemia is consistently associated with worse outcomes following open abdominal surgery, including higher risks of wound-related complications (e.g., dehiscence) and mortality [53]. In inguinal hernia surgery, malnutrition and low albumin predict worse outcomes after open or laparoscopic repair, with complications and adverse events rising as albumin decreases [54].

#### Micronutrients

Deficiencies of vitamins C and A, zinc, and copper may impair central wound-healing processes, including collagen synthesis and remodeling, epithelial growth/reepithelialization, and immune response. Vitamin C is a cofactor for prolyl and lysyl hydroxylases essential for collagen hydroxylation and stabilization; vitamin A supports epithelial growth and fibroblast activity; zinc contributes to reepithelialization, tissue formation, and immune function; and copper participates in multiple wound-healing phases. Together, these deficiencies may delay tissue repair and compromise the quality of healing, with potential negative effects on the integrity of surgical repair [55, 56].

#### Immunonutrition

In AWH surgery, immunonutrition may be considered within perioperative optimization for patients with greater surgical complexity or nutritional vulnerability. ESPEN guidelines support nutritional screening and perioperative nutritional therapy in surgical patients at nutritional risk, including immunonutrient-enriched formulas in selected contexts, while acknowledging heterogeneous evidence and uncertainty regarding optimal candidates and timing [13]. Recent AWH perioperative protocols also include pre- and postoperative immunonutrition as a consensus variable, and evidence from major abdominal surgery suggests reduced overall morbidity, although effects on mortality, infectious complications, and length of stay remain uncertain [57, 58].

### Digital tools

AI and ML are increasingly used in surgery for risk prediction and decision support based on perioperative data. In abdominal wall reconstruction, a multicenter study developed preoperative CT-based deep learning models to estimate (i) surgical site infection (SSI) risk and (ii) a proxy for surgical complexity, defined as the probability of requiring a component separation technique, showing the feasibility of such tools for risk prediction [59]. Another study suggests that federated learning can train robust models to predict postoperative complications using multicenter data without centralizing sensitive data, supporting privacy-focused collaboration [60]. A deep learning model with dynamic, time-varying predictions showed that high-resolution intraoperative physiological signals can improve prognostication of postoperative complications [61].

## Discussion

Traditional AWH management remains challenging over the long term because recurrence is cumulative and varies substantially by hernia type. In a national analysis of prospectively recorded Danish Ventral Hernia Database data, reoperation for recurrence continued to rise even after 15 years; at 15 years, reoperations for recurrence were 10.5% after umbilical hernia repair, 14.3% after epigastric repair, approximately 18–20% after incisional repairs, and approximately 40% after parastomal hernia repair [62]. Because this outcome captures only reoperated patients, the “true” recurrence rate is probably higher than the reoperation-based estimate [62].

Mechanically and biologically, reviews of abdominal wall and biomaterial properties highlight the heterogeneity of tissue characteristics (dependent on local anatomy) and a fundamental gap in understanding how much mechanical mismatch between host tissue and prosthesis contributes to interface failures and late poor outcomes [19]. Together, these findings reinforce that recurrence remains a cumulative outcome and that advancing care requires, beyond technique, a deeper understanding of abdominal wall biomechanics and how biomaterials interact with tissues that differ among patients [19, 62].

In patient subgroups, AWH may be better understood as a complex phenotype in which connective tissue and ECM factors, particularly collagen remodeling and inflammation, interact with mechanical and clinical determinants. A recent review consolidates genetic and biological factors in hernia formation and discusses ECM and collagen turnover pathways as plausible mechanisms [9]. Accordingly, AWH may represent a local manifestation of systemic ECM impairment, with systemic and local alterations in collagen metabolism and turnover across hernia presentations supporting a systemic contribution to pathophysiology [63, 64]. This finding reinforces the rationale for considering interindividual heterogeneity and underlying biology in therapeutic planning.

Genomic evidence supports a polygenic architecture for AWH, with GWAS identifying susceptibility loci across hernia phenotypes, including connective-tissue-related signals and more than 80 loci in larger analyses [12, 47, 65, 66]. Complementary TWAS data suggest shared pleiotropy among hernia subtypes and link genetic predisposition to tissue-related effector pathways [42]. Although these findings support future exploration of variants or polygenic scores in risk models, clinical translation requires standardized phenotypes, robust outcome definitions, and external validation; multi-omic Mendelian randomization may further generate causal hypotheses and prioritize biological targets for functional validation [9, 12, 42, 65–67].

Clinical studies and systematic reviews also support systemic ECM remodeling and collagen turnover. A systematic review of 14 studies concluded that ECM remodeling and collagen metabolism markers are among the most promising circulating biomarkers, despite methodological heterogeneity and the need for validation [20]. The serum collagen turnover profile differs between patients with inguinal and incisional hernias and controls, with biomarkers reflecting synthesis and degradation of interstitial matrix and basement membrane collagens [30]. Reviews of collagen turnover in hernias reinforce the need for analytical standardization, phenotype definition, and validation in larger cohorts [64].

In inguinal hernia, studies evaluating blood MMPs and procollagen propeptides support that circulating biomarkers may reflect an altered balance between ECM synthesis and degradation [25]. More recently, a study of circulating biomarkers in inguinal hernia simultaneously measured enzymes and propeptides related to collagen metabolism (including LOX, PINP, PIIINP, and PIVNP, as well as MMP-2 and MMP-9) and reported associations with the presence of hernia and discriminatory performance in case-control analyses, discussing their potential as candidate markers to compose stratification strategies [26]. Circulating markers of type III collagen turnover (such as PIIINP/P3NP) are also associated with aging-related functional outcomes in population cohorts, reinforcing that they may capture systemic processes relevant to predictive models [68]. This supports serum phenotyping as a component of risk stratification; however, reviews highlight methodological heterogeneity and the need for analytical standardization, phenotypic definition, and prospective validation in larger cohorts [20, 30]. Mendelian randomization approaches have also prioritized biological targets for postoperative incisional hernia risk, offering complementary hypotheses for future validation [67].

Current evidence supports an association between inflammatory–stromal dysregulation and abdominal wall hernia, although causality remains unproven. Incisional-hernia tissues demonstrate compartment-specific fibroblast phenotypes, while inguinal-hernia specimens and complementary skin-fibroblast experiments implicate IL-1β–p38 MAPK–MMP-2 signaling in impaired cell proliferation, migration, and collagen remodeling [34, 35]. Mendelian randomization studies provide complementary but heterogeneous evidence linking selected cytokines and immune-cell traits to hernia susceptibility or consequence [38, 39]; however, small tissue cohorts, established-disease sampling, overlap between explant studies, selection bias, and genetic-inference assumptions preclude definitive causal conclusions. Mesh implantation should be considered a material- and context-dependent modifier of repair rather than a host biomarker. Synthetic polymers initiate protein adsorption followed by innate and adaptive immune-cell recruitment, macrophage and foreign-body giant-cell responses, fibroblast activation, collagen deposition, and variable fibrotic encapsulation, the organization of which is influenced by biomaterial properties [69]. In seven human polypropylene meshes explanted for recurrence, macrophages and T-cell subsets were concentrated near the fibers, while collagen I/III deposition and MMP-2 expression accompanied an M2-marker-predominant response with substantial phenotypic overlap [36, 37].

Perioperatively, modifiable factors should inform surgical risk discussions. ESPEN guidelines recommend systematic nutritional risk screening and perioperative nutritional therapy when indicated to reduce complications and promote recovery, reinforcing nutritional status as a relevant dimension of surgical care [13]. In complex abdominal wall surgery, imaging-assessed sarcopenia has been investigated as a marker of host vulnerability associated with worse postoperative outcomes, supporting the inclusion of body composition and functional status in perioperative care [14]. In AWH, combining albumin and prealbumin with body composition (e.g., CT-based sarcopenia) and comorbidities may improve risk stratification for wound complications and recovery.

Imaging phenotyping adds an objective layer for capturing morphological and anatomical vulnerabilities of the abdominal wall. Preoperative CT studies identified morphological features associated with incisional hernia after abdominal surgery [70] and proposed “optimized” CT biomarkers for predicting incisional hernia by refining and selecting morphometric measures [71]. Shear wave elastography quantified muscle properties in incisional hernia, suggesting that stiffness and thickness measurements capture biomechanical aspects relevant to planning and stratification [72]. Together, these imaging approaches may link tissue predisposition to the anatomical expression of the hernia phenotype [70–72].

AI applied to the abdominal wall already shows utility in imaging-derived prediction. In abdominal wall reconstruction, one study developed and validated preoperative CT-based deep learning models that predict surgical complexity (need for component separation) and estimate complication risk, including SSI, supporting automated models for planning and risk assessment [17]. Recent reviews of AI/ML in hernia surgery describe the field’s progress and organize the main applications into (i) prediction of outcomes and complications, (ii) automated image analysis, and (iii) perspectives for decision support and surgical workflows [18, 73, 74]. Although contemporary literature provides consistent signals for a more complex biological basis of AWH, polygenic predisposition, serum ECM remodeling biomarkers, quantitative imaging metrics, and AI-based prediction, no integrated, externally validated multimodal model yet combines these domains to estimate risk of formation, recurrence, and complications.

### Future perspectives

Future perspectives in precision herniology should focus on the validated integration of tissue biology, circulating biomarkers, artificial intelligence, quantitative imaging, advanced biomaterials, and digital-twin concepts into reproducible translational frameworks. Fibroblast phenotyping and plasma proteomics may help identify biological subgroups and candidate biomarkers for incisional hernia risk, while machine learning, deep learning, shear wave elastography, and electrospun meshes may support risk prediction, biomechanical characterization, and more tailored abdominal wall repair [73–76]. In parallel, digital twins, living-lab models, and real-world data ecosystems could enable individualized simulation, continuous model refinement, and multicenter evaluation, provided that interoperability, data governance, privacy, algorithmic transparency, and ethical implementation are rigorously addressed before clinical adoption [77–82] (Table 2).

**Table 2:** Future directions for precision herniology

Research domain	Data layers	Future application
Collagen and ECM turnover	PINP, PICP, PIIINP, ICTP, NTX, C1M, C3M, MMPs, and TIMPs	Preoperative biological risk stratification for recurrence, wound complications, impaired healing, and mesh integration
Cellular and inflammatory phenotype	Fibroblast marker profiles and MMP expression; macrophage and lymphocyte populations at the mesh–tissue interface; local cytokine and growth-factor signaling	Identification of hosts with a catabolic or poorly resolving repair phenotype; interpretation of circulating markers in cellular context
Emerging serum omics	Glycomic, proteomic, metabolomic, and lipidomic signatures	Identification of systemic biological phenotypes beyond isolated collagen markers
Quantitative imaging and biomechanics	CT morphometrics, abdominal wall geometry, muscle quality, sarcopenia, and elastography-derived stiffness	Patient-specific assessment of anatomical and biomechanical vulnerability
Mesh and material personalization	Host biology, defect morphology, tissue stiffness, inflammatory risk, and ECM profile	Transition from one-size-fits-all repair to host-adapted abdominal wall reconstruction
Multimodal digital model of the abdominal wall	Integrated clinical, biomarker, imaging, biomechanical, operative, and longitudinal data	Research platform for individualized simulation, dynamic risk updating, precision surgical planning and mesh selection
*ECM* extracellular matrix, *AI *artificial intelligence, *CT *computed tomography		

### Limitations

As a critical narrative review, this manuscript is subject to selection, interpretation, and publication bias and was not designed to quantify effects, compare interventions, or provide the methodological exhaustiveness of a systematic review. Moreover, much of the available evidence derives from heterogeneous populations, AWH subtypes, biological matrices, analytical platforms, outcomes, and follow-up durations, limiting comparability and direct extrapolation. The cellular, inflammatory, and host–prosthesis evidence warrants particular caution: human tissue studies are small and cross-sectional, predominantly sampling established or recurrent disease, and partly rely on cultured skin fibroblasts or murine models rather than human fascia. Mesh-explant data are derived from prostheses removed because of complications and cannot represent uncomplicated integration, whereas macrophage phenotyping relies on limited marker panels within an M1/M2 dichotomy that oversimplifies the phenotypic continuum. Consequently, no cellular, inflammatory, or biomaterial-related signature has been prospectively linked to recurrence, wound morbidity, or mesh integration, and none is currently suitable for clinical risk stratification or individualized prosthetic selection.

The domains integrated here also have unequal analytical and translational maturity. Although some markers offer consistent mechanistic plausibility, their specificity for the hernia phenotype and incremental value over conventional clinical variables remain uncertain. Likewise, extrapolation among inguinal, primary ventral, incisional, and complex reconstructive scenarios warrants caution, because the relative weight of biological, mechanical, and perioperative determinants may vary substantially across contexts.

The proposed framework should therefore be interpreted as a conceptual structure and translational validation agenda, not a clinical tool ready for implementation. Its translation will depend on prospective multicenter cohorts, phenotypic and analytical standardization, external validation, and demonstrated incremental utility over current risk models.

## Conclusions

Available evidence supports the hypothesis that host biological heterogeneity contributes to AWH formation, healing, recurrence, and prosthetic integration. The main value of this review is the integration of ECM biomarkers, cellular and inflammatory mechanisms, genetics, immunonutritional vulnerability, quantitative imaging, biomaterial–host interactions, and AI/ML within a single translational framework. The next leap in abdominal wall surgery may lie not only in better defect repair but also in recognizing, before operation, which hosts carry greater biological vulnerability to repair failure.

## Supplementary Information

Below is the link to the electronic supplementary material.

Supplementary Material 1

Supplementary Material 2

Supplementary Material 3

Supplementary Material 4

Supplementary Material 5

Supplementary Material 6

## Data Availability

All data generated or analyzed during this study are included in this published article.
